# Knowledge, Attitudes, and Practices of Bedside Nurses regarding Antimicrobial Stewardship in China: An Explanatory Sequential Mixed Methods Study

**DOI:** 10.1155/2023/9059920

**Published:** 2023-11-22

**Authors:** Wenting Zhao, Yufei Xu, Ru Liu, Tingting Zhao, Yan Ning, Yaoqing Feng, Hongyun Kang, Shuhua Li, Xiaojuan Han, Linping Shang

**Affiliations:** ^1^Nursing College, Shanxi Medical University, Taiyuan 030001, China; ^2^Department of Nursing, First Hospital of Shanxi Medical University, Taiyuan 030001, China; ^3^Department of Nursing, Changzhi Medical College, Changzhi 046000, China; ^4^Nursing Department, Shanxi Bethune Hospital, Taiyuan 030032, China; ^5^Drug Clinical Trial Organization, Heping Hospital of Changzhi Medical College, Changzhi 046000, China; ^6^Department of Public Health, Shanxi Medical University, Taiyuan 030001, China

## Abstract

**Aim:**

To evaluate the knowledge, attitudes, and practices of Chinese bedside nurses regarding antimicrobial stewardship, as well as to identify factors that influence nurses' engagement in antimicrobial stewardship.

**Background:**

Antimicrobial resistance is a pressing global health threat. Antimicrobial stewardship is crucial in combating this issue. Nurses play a key role in implementing antimicrobial stewardship. However, little is known about the involvement of Chinese nurses in antimicrobial stewardship.

**Methods:**

An explanatory sequential mixed-methods design was employed. A self-developed questionnaire was administered between March and August 2021 (*N* = 463), followed by semi-structured interviews with 17 nurses between March and July 2022. Descriptive statistics and qualitative content analysis were used to analyze the data.

**Results:**

The study found that nurses scored 75% in knowledge, 82.8% in attitude, and 84.1% in practice domains. There was a moderate correlation between nurses' knowledge, attitudes, and practices. It revealed that knowing local antimicrobial stewardship programmes information and the frequency of receiving antimicrobial stewardship training had a significant impact on nurses' knowledge scores. The willingness to participate in related training influenced nurses' attitude scores. Being a clinical teacher and the frequency of receiving related training influenced nurses' practice scores. The qualitative phase identified three themes: insufficient knowledge of nurses' engagement, diverse attitudes towards engagement, and limited scopes and absence of standards in nurses' participation.

**Conclusions:**

Our findings emphasize the importance of enhancing nurses' perception and involvement in antimicrobial stewardship. While nurses exhibit positive attitudes and practices, addressing the existing knowledge gap is crucial. To achieve this, it is necessary to clarify the role and responsibilities of nurses in antimicrobial stewardship, provide regular training and innovative methods, strengthen communication and collaboration, and foster a positive work environment. Additionally, actively promoting the development of guidelines and evaluation criteria will enable nurses to more effectively participate. *Implications for Nursing Management*. Regular training of nurses in antimicrobial stewardship needs to be enhanced. Nursing managers should strive to create positive, empowering, and supportive work environments, participate in policy formulation and implementation, and provide clear expectations for nurses' engagement in antimicrobial stewardship.

## 1. Introduction

The rapid increase in antimicrobial resistance (AMR) poses a serious threat to global health [1]. According to an AMR review commissioned by the United Kingdom government, AMR will cause 10 million deaths a year and cost $1 trillion annually by 2050 [2]. China, as one of the largest consumers of antimicrobials worldwide, faces significant challenges in combating AMR [3]. Due to its high antibiotic consumption rate, China plays a crucial role in the global burden of AMR. In fact, between 2000 and 2015, antimicrobial consumption in China witnessed a staggering 79% increase, surpassing the global average increase in antimicrobial consumption [4]. The existing evidence indicates that the inappropriate use and misuse of antimicrobials are major drivers of increasing AMR [3, 5, 6]. To effectively control and prevent AMR, the involvement of healthcare professionals, particularly nurses, in antimicrobial stewardship (AMS) is crucial [7].

AMS is a coherent set of actions that promote the use of antimicrobials in ways that ensure sustainable access to effective therapy for all who need it [5]. It is a rapidly growing field in medicine with the goal of rational use of antibiotics in terms of dosing, duration of therapy, and route of administration [8]. Furthermore, it is a multidisciplinary approach involving various healthcare professionals, such as microbiologists, pharmacists, infection control practitioners, physicians, and epidemiologists, who are dedicated to controlling AMR [9].

Extensive research has provided compelling evidence regarding the vital roles that nurses play in communication and AMS [10, 11]. These roles encompass prompting, reminding, checking, and questioning prescribers' decisions, all of which contribute to the appropriate use of antimicrobials. Furthermore, with nurses representing the largest healthcare workforce worldwide, their involvement in the comprehensive AMS holds immense potential [6, 12], especially in monitoring the selection, timing, and duration of antimicrobial use, as well as dosage administration [12]. Even a marginal increase of 1% in their engagement, utilizing their full clinical and leadership potential, can yield substantial benefits for healthcare services and patients [13]. It is particularly beneficial in settings where human resources are scarce and it is difficult to establish AMS teams formed by physicians and pharmacists [13].

Several studies [14–16] have investigated the involvement of nurses in AMS, focusing on their knowledge, attitudes, and practices (KAP). These studies found that nurses had a restricted comprehension of the significance of AMS and their corresponding role within it. In addition, they lacked awareness about their specific responsibilities and the particular nursing practices associated with AMS [16, 17]. This lack of awareness poses challenges to fully utilizing nurses' potential in AMS initiatives [18, 19]. Currently, there is no study illustrating the KAP about AMS among nurses in China. A recent panel survey from China even revealed that a larger number of nurses was associated with poorer AMS performance [20], highlighting the need for further investigation and interventions to address any barriers or gaps in knowledge, attitudes, and practice among nurses in China.

This study aims to investigate Chinese nurses' KAP related to AMS and identify the factors influencing their participation. To comprehensively understand these aspects, an explanatory sequential mixed-methods design, combining quantitative and qualitative approaches, is employed. The quantitative phase provides initial insights and trends, while the subsequent qualitative phase offers a deeper understanding and explanation of the observed results [21]. This sequential approach enhances the validity and reliability of the findings and allows for the exploration of outliers, surprising results, and positive-performing exemplars [21]. By employing this mixed-methods design, this study aims to capture the complexity of nurses' perspectives on AMS and the factors influencing their participation. Guided by pragmatism [21], which emphasizes practical knowledge and real-world application, this study offers practical insights for local initiatives involving nursing in AMS. The findings will contribute to both local and global strategies for combating AMR, identifying common challenges and effective approaches in diverse healthcare settings.

## 2. Methods

This study employed an explanatory sequential mixed-methods design [21], which involves collecting and analyzing quantitative data followed by qualitative data to provide further clarification and supplementation to the quantitative findings. The theoretical framework that guided this study is the Knowledge, Attitudes, and Practice (KAP) model proposed by British scholar Kirster [22]. This model divides the process of behaviour change into three continuous processes: acquiring knowledge, generating beliefs, and forming behaviours [23]. The KAP model was used to guide the survey framework and analysis in the quantitative phase and as an interpretive lens to frame the themes in the qualitative phases.

The quantitative phase of the study had a cross-sectional design and involved the administration of an anonymous online questionnaire between March and August 2021. The questionnaire was designed to assess nurses' knowledge, attitudes, and practices related to AMS. To gain a comprehensive understanding of the factors influencing nurses' engagement in AMS, a qualitative descriptive research design was employed. This involved conducting individual semistructured interviews with nurses between March and July 2022.

To ensure methodological rigour, the study adhered to the guidelines outlined in the Strengthening the Reporting of Observational Studies in Epidemiology (STROBE) statement and Consolidated Criteria for Reporting Qualitative Research (COREQ) (see Supplementary File 1 and File 2). In addition, the entire study adhered to the Good Reporting of a Mixed Methods Study (GRAMMS) criteria (Supplementary File 3).

### 2.1. Setting

The study was conducted in three tertiary hospitals in northern China, selected based on their long-term implementation of AMS programmes and their status as the largest medical facilities in the region with 3,000, 2,700, and 2,270 beds, respectively. The number of registered nurses at these hospitals was 1,846, 1,750, and 1,500, respectively.

### 2.2. Participants and Sampling

In the quantitative phase, the sample size was determined using Raosoft's online sample size calculator (https://www.raosoft.com/samplesize.html), with a confidence interval of 95%, a margin of error of 5%, and an assumed response distribution of 50% [8]. Considering a potential attrition rate of 20% and the possibility of outliers, a minimum sample size of 453 was deemed necessary. The inclusion criteria were defined as follows: registered nurses with assigned patient care responsibilities and a minimum employment duration of 6 months. Nursing interns and nurses who were registered in other healthcare institutions but undergoing short-term training at the three hospitals were excluded.

In the qualitative phase, purposive sampling was used to select participants who could provide unique and insightful information to uncover new knowledge systems [24]. Seventeen nurses were invited to participate based on their characteristics, knowledge, and expertise. Participants were selected from various departments, organisational levels, and played different roles. The inclusion criteria were as follows: (a) willingness to participate, (b) being a registered nurse involved in direct patient care, (c) having at least 3 years of work experience, and (d) participation in the first-stage questionnaire survey. The exclusion criteria for this phase were consistent with those used in the quantitative phase.

Recruitment was primarily conducted through announcements on WeChat Workgroups, and a snowballing approach was used where interviewees recommended other eligible and interested candidates to the researchers. Recruitment was terminated when data saturation was reached, defined as the point at which three consecutive interviews failed to yield any new information [25].

### 2.3. Data Collection

#### 2.3.1. The Quantitative Phase

The data for the quantitative phase were collected using the Wenjuanxing online survey platform, which is widely used in China. An online questionnaire was created on the platform, and a letter explaining the purpose, significance, and survey process of the study was sent to the hospital administrators of each participating hospital to obtain permission for data collection. Once administrative approval was obtained, the researchers contacted the nurse managers of the respective wards. The nurse managers introduced the project background and the inclusion and exclusion criteria for the research subjects in the WeChat workgroup, and then released the questionnaire. As the survey was anonymous, completion of the questionnaire implied consent.

The questionnaire consisted of 51 items divided into four sections. These items were developed based on a literature review [26] and underwent further refinement through group discussions. The split-half reliability coefficient for this questionnaire was 0.783, the Cronbach's alpha reliability coefficient for the overall questionnaire was 0.964, and the overall content validity index was 0.90, all of which were validated in a pilot study [27].

The first section of the questionnaire included seven demographic items and three prior experience questions. Demographic items included age, sex, education level, years of nursing experience, and experience of AMS education. The second section comprised 15 items to assess participants' AMS knowledge, using a 5-point Likert scale ranging from strongly disagree (1) to strongly agree (5). The section covered topics such as the purpose and team structure of AMS (2 items), participants' understanding of antimicrobial usage (7 items), and participants' AMR-related knowledge (6 items). To assess nurses' attitudes towards AMS, the third section employed a 5-point Likert scale consisting of 14 questions, which explored nurses' perspectives on participation in AMS programmes (5 items), safe and proper administration of antimicrobials (6 items), and the reduction of exposure to antimicrobials (3 items). The final section included 12 questions that assessed nurses' adherence to AMS practices, using a Likert scale ranging from 1 (never) to 5 (always). Questions in this section covered topics such as diagnostic management assistance practice (2 items), proficiency in safe administration techniques for antimicrobials (5 items), and participation in AMS programmes (5 items).

#### 2.3.2. The Qualitative Phase

In the qualitative phase, face-to-face semistructured interviews were conducted to gather comprehensive insights into nurses' experiences, perceptions, and factors influencing their participation in AMS initiatives. To address the issue of insufficient knowledge identified in the quantitative findings, specific questions were included in the interview guidelines. For example, nurses were asked for their opinions on the finding that many nurses felt they lacked adequate knowledge of AMS. In addition, attitudes towards AMS were further examined by incorporating questions that built upon the positive attitudes identified in the quantitative phase. For instance, nurses were asked to provide their thoughts on the finding that most nurses were willing to participate in AMS, as well as any reasons for potential hesitation. To gain deeper insights into the practical aspects of AMS, questions were developed to explore nurses' experiences and perceptions related to specific AMS practices they were involved in, such as discussions about antibiotic de-escalation. These questions aimed to provide a more nuanced understanding of the practice-setting issues identified in the quantitative phase. The final interview list can be found in Supplementary File 4. It is important to note that not all questions from the interview list were posed during each interview. The selection and sequencing of questions were determined by the natural flow of the conversation and the interviewee' responses. This approach allowed for a more organic and flexible interview process, ensuring that the questions asked were relevant and responsive to the specific context and insights provided by each interviewee [28].

Two nurses were purposively selected for preinterviews, and based on the initial interviews, the guidelines were further refined. The interviews were conducted by a primary interviewer (WT Z) and a note-taker (YF X) in either the department office or nurses' lounge, based on the interviewees' preferences. Written consent was obtained from the interviewees before the interviews, which were conducted in Standard Mandarin. With permission from the interviewees, conversations were digitally recorded. The interview questions were open-ended, allowing the interviewees to freely express their perspectives. The interviewers maintained a neutral demeanour throughout the interviews to ensure that the interviewees' responses were not influenced or coerced. The average duration of each interview was between 30 and 50 minutes. Data saturation was reached after 14 interviews, with an additional three interviews conducted for confirmation purposes. Two researchers (WT Z and YF X) reviewed the interviews to ensure comprehensive coverage of the research question and confirmed the adequacy of the sample size. Three follow-up telephone calls were conducted with three participants to supplement and clarify certain details, and no additional formal interviews were scheduled or conducted. All the researchers processed prior experience and training in qualitative research methods.

### 2.4. Data Analysis

The survey data collected in the quantitative phase were analyzed using IBM SPSS Version 23.0 (Armonk, New York, USA: IBM Corp). Descriptive statistics such as means, standard deviations, frequencies, and percentages were used to describe the distributions of the variables. The scoring rate (RT) was calculated by dividing the total score (or score of each domain) by the maximum possible score (or maximum possible score of each domain), and then multiplying by 100% [29]. RT ≥ 80% (4/5) indicated a good level of knowledge, attitudes, and practices regarding AMS, while RT < 80% indicated poor performance in AMS [30].

To examine the differences in participants' knowledge, attitudes, and practice levels, independent sample *t*-tests, analysis of variance, and multiple linear regression analysis were conducted. In addition, correlation analysis was performed to explore the relationships between participants' knowledge, attitudes, and practice levels.

The audio recordings in the qualitative phase were fully transcribed verbatim by a researcher (WT Z) within 24 hours after each interview and reviewed by one researcher (YF X) for accuracy. The transcripts were then returned to the participants for their input, allowing them to provide any comments or corrections. Finally, the transcripts were securely stored in password-protected Microsoft Office Word documents, guaranteeing the confidentiality and integrity of the data.

The software NVivo V.12 (QSR International, Doncaster, Australia) was employed for organising and coding the data. A content analysis approach with a three-stage process was used to analyze the qualitative data [31]. In the preparatory stage, we recorded the interviews and transcribed them verbatim to ensure accuracy. Descriptive summaries were immediately provided after each interview and shared with the research team. To familiarize ourselves with the data, two researchers (WT Z and YF X) repeatedly read the transcripts and descriptive summaries. In the preparatory phase of the analysis, the data were categorised and deductively coded, and the codes were combined based on the relevant themes (interviewees' knowledge, attitudes, and practice) that were identified and guided the coding process. In the organisational phase, we identified potential categories, patterns, and themes. The interviewers (WT Z and YF X) led the process of open coding and discussed the identification of codes, patterns, and themes during weekly team meetings. The data was classified and reclassified into comprehensive, higher-order themes until consensus was reached among the research group members. A third researcher (LP S) reviewed the initial analysis and compared the coding to ensure the consistency and interpretability of the data. Finally, we shared the results with all participants and requested their feedback or opinions to be incorporated into the report of the study findings. In the reporting phase of the analysis, direct quotes from interviewees were used to describe and substantiate each theme.

To ensure the privacy and confidentiality of the interviewees, their names were replaced with serial numbers. Data analysis was performed in Chinese to prevent any misunderstandings. During the drafting of the article, the final findings were translated into English by a professional translator fluent in both English and Chinese. This ensured the grammatical accuracy, fluency of the translated text, and accuracy of the translation itself. In addition, no funding was received for this study, and the researchers had no prior bias or specific assumptions about the research themes.

To ensure the rigour and trustworthiness of this phase, the four criteria of Lincoln and Guba for qualitative research were applied [32, 33]. For credibility, the information collected from the interview recordings, the codes applied by both researchers, and the responses reviewed by the interviewees were compared to identify coherence and discrepancy in the utterances. To establish dependability, memos were written after each interview, documenting the interviewees' nonverbal language, willingness to participate, and other contextual details. To enhance transferability, rich descriptions and direct quotes from interviewees were provided to exemplify the identified themes. Moreover, confirmability was ensured through journaling, where a record of our thoughts, biases, and preconceptions was maintained. This practice of reflexivity helped acknowledge and mitigate potential biases.

### 2.5. Data Integration

The findings from the quantitative stage were compared and interpreted alongside the findings from the qualitative phase using joint display analysis. All authors participated in discussions throughout this integration process.

### 2.6. Ethical Considerations

The study protocol adhered to the principles of the Declaration of Helsinki, and permission for the study was obtained from the research ethics committee of the First Hospital of Shanxi Medical University. In the quantitative phase, participants were informed through a letter accompanying the questionnaire that their participation was voluntary and would have no effect on their work and life. Participants' completion and return of the questionnaire were considered to be their informed consent. In the qualitative phase, all interviewees provided signed informed consent before participating in the interviews. Ethical approval included consent for participation and potential publication.

## 3. Results

### 3.1. Quantitative Findings

#### 3.1.1. Characteristics of the Study Population

A total of 470 nurses responded to the online survey, but seven were excluded due to concerns regarding quality control (all questions had identical responses). Most respondents (*n* = 432, 93.3%) were women and were between 20 and 40 years old. Approximately 61.6% of nurses (*n* = 285) had a primary professional title, and 96.5% (*n* = 447) had a bachelor's degree or higher. Although we purposefully selected three tertiary hospitals which had AMS programmes, 31.5% (*n* = 146) of nurses were unaware of the existence of AMS teams in their hospitals. Furthermore, 178 nurses (38.4%) had not received AMS training in the past year, and over 430 nurses (92.9%) expressed interest in participating in AMS training.

#### 3.1.2. Knowledge, Attitudes, and Practices of Nurses regarding AMS

The mean knowledge score of the respondents was 56.24 (standard deviation, SD = 9.79), and the scoring rate was 75.0% (lower than a set standard of 80%), indicating that nurses' knowledge of AMS was insufficient. Among the participants, 197 nurses (42.6%) were unaware of the importance or purpose of AMS, and 38.4% of nurses expressed uncertainty or a lack of understanding regarding the indications for antimicrobial therapy in asymptomatic bacteriuria patients.  Table 1 presents the three lowest and highest-scoring items for the knowledge domain. Additional detailed information on all items of nurses' KAP on AMS can be found in Supplementary Table 1.

By contrast, the overall mean attitude score was 57.94 (SD = 8.81), and the scoring rate was 82.8% (TS = 70), indicating that the nurses' attitudes towards AMS were positive (higher than a set standard of 80%). A total of 378 respondents (81.6%) agreed that nurses should participate in AMS, and 384 respondents (82.9%) agreed that nurses should be positive about acquiring up-to-date knowledge regarding antimicrobials and AMR. See Table 2 for the three lowest and highest items of the attitudes domain.

Nurses' performance in AMS practices (mean score 50.4 ± 7.51) had an overall scoring rate of 84.1% (TS = 60). The top three items with the highest and lowest scores are shown in Table 3.

#### 3.1.3. Factors Related to Knowledge, Attitudes, and Practices of Nurses

*(1) Univariate Analysis*. The study conducted a univariate analysis to examine the relationship between the dependent variables (knowledge, attitude, and practice scores) and the independent variables (demographics and experience of AMS). The analysis revealed that several factors had a significant impact on nurses' knowledge of AMS, including age, professional title, clinical teaching status, knowledge of the existence of AMS programme in the hospital, willingness to undergo AMS training, years of nursing experience, frequency of receiving AMS education in the past year, and the department where they worked (*P* < 0.05). The detailed results of the analysis can be found in Table 4.

*(2) Multivariate Analysis*. The multiple linear regression analysis revealed that a significant independent factor influencing nurses' knowledge, attitudes, and practices regarding AMS was the frequency of receiving AMS education during the past year (*P* < 0.05). Although the univariate analysis indicated that age, professional title, nursing experience, and department were associated with a change in KAP scores, the multivariate analysis did not support this finding (Table 5).

*(3) Correlation Analysis of Knowledge, Attitudes, and Practices of Nurses*. According to Pearson correlation analysis, nurses' knowledge and attitudes, attitudes and practices, and knowledge and practices regarding AMS were moderately positively correlated, with correlation coefficients of 0.510 (*R*^*2*^ = 0.260), 0.555 (*R*^*2*^ = 0.323), and 0.569 *(R*^*2*^ = 0.309), respectively (*P* < 0.001).

### 3.2. Qualitative Findings

A total of 17 nurses participated in semi-structured interviews. The majority of the nurses were female (94.12%), and their ages ranged from 26 to 48 years. The duration of their nursing experience varied from 3 to 28 years, and they worked in different departments. More detailed characteristics of the interviewees can be found in Supplementary Table 2. From the interviews, three main themes emerged regarding nurses' engagement in AMS: insufficient knowledge, diverse attitudes, limited scopes, and the absence of standards in their participation in AMS practices.

#### 3.2.1. Theme 1: Insufficient Knowledge of Nurses' Engagement in AMS

Insufficient AMS-related knowledge is a major barrier to nurse engagement in AMS. A significant proportion of nurses (8 out of 17) were unaware of what AMS is, and five nurses mentioned that AMS was primarily the responsibility of doctors and pharmacists, with little relevance to nursing. Three interviewees reported a lack of understanding regarding the specific content and scope of nursing practice in the context of AMS.

*(1) Sub-Theme 1: Limited Opportunities to Engage in AMS Training*. The interviewees reported that AMS training in their hospitals primarily focused on doctors, pharmacists, and laboratory technicians, with less emphasis placed on involving nurses. Even when nurses were notified about training opportunities, they often faced challenges related to time constraints and scheduling conflicts, making it difficult for them to actively participate in the training.

*I have rarely received this kind of AMS training. Generally, this training is specifically designed for doctors. Even if I want to participate, I don't know when and where AMS training will be held. (N11)*

*This training is mostly scheduled during working hours, which presents a challenge for us nurses. Our workload is already overwhelming, making it difficult to find the time to attend. Even when there are training sessions during break time, we often feel exhausted and lack the energy to fully engage. (N8)*

*(2) Sub-Theme 2: Limited Opportunities to Apply and Retain AMS Knowledge*. Despite receiving education on antibacterials during their degree courses or continuing education, nurses frequently encounter difficulties in effectively applying and retaining this knowledge in their daily practice. The demanding and fast-paced work environment often left nurses with insufficient time to fully consider and apply their acquired knowledge, leading to a gradual loss of important details over time.

*We often find ourselves lacking the necessary time and opportunities to fully delve into our knowledge, resulting in the gradual loss of important details. For example, when things become busy, it becomes challenging to monitor patients' conditions as meticulously as we were trained to do. As time goes by, this knowledge gradually fades away. (N7)*

Furthermore, the restricted input from nurses in prescription decisions further contributed to this perception. This limitation leads to a dearth of opportunities for nurses to apply their AMS knowledge in practical settings.

*We are seldom present during multi-disciplinary rounds, and even if we do attend, we lack the authority to provide input on prescription decisions. (N10)*

#### 3.2.2. Theme 2: Diverse Attitudes towards Engagement in AMS

The interviewees had diverse attitudes towards engagement in AMS training and practice. A positive attitude primarily stemmed from the recognition of the significance of AMS and a sense of responsibility toward patients safety. Conversely, a negative attitude mainly originated from doctors' sensitivity to their authority, as well as the fear of being blamed. Additionally, department cultures, including doctor-nurse relationships and peer attitudes, could influence nurses' confidence in participating, consequently impacting their attitudes towards AMS involvement.

*(1) Sub-Theme 1: Diverse Attitudes towards Engagement in AMS Training*. Most interviewees recognised the importance of participation in AMS and tend to have a more positive attitude towards AMS training. In addition, nurses who demonstrate a strong sense of responsibility towards patient safety exhibit a commendable commitment to engaging in AMS training, even in the face of acknowledging their demanding workload and limited time and energy.

*I am highly motivated to learn about AMS because it is our duty and a crucial aspect of our work. Acquiring knowledge in this area enables us to identify and prevent potential microbial resistance crises, ultimately reducing the difficulties and losses experienced by our patients. (N3)*

However, five nurses held the view that AMS is predominantly the domain of doctors and pharmacists, perceiving it as having minimal relevance to their nursing duties. This perception further dampens their motivation to engage in AMS training.

*I don't believe that the training holds much practical value for our work as nurses. After all, prescribing and making choices about antibiotics are not typically included in our job description. (N13)*

*(2) Sub-Theme 2: Diverse Attitudes towards Engagement in AMS Practice*. Nurses demonstrated a positive attitude towards participating in AMS to their strong sense of responsibility, patient safety awareness, and the presence of a harmonious doctor-nurse relationship. Those who expressed a sense of responsibility in advocating for patient rights displayed a commendable willingness to actively engage in AMS practice.

*It's crucial to the treatment and recovery of patients, and I think our participation in AMS can better facilitate collaboration with doctors, as well as better safeguard the interests and safety of patients. (N12)*

*That's the way we actively work to protect our patients' rights and keep them safe. It's also a big part of our job. (N6)*

The confidence of nurses in participating in AMS is largely influenced by the doctor-nurse relationship. In healthcare settings where doctors and nurses enjoy a harmonious relationship, nurses are more inclined to seek information from doctors regarding AMS and feel encouraged to present their suggestions and actively participate in decision-making processes.

*If this situation occurs in a good doctor-nurse relationship, doctors will be very patient, and we are willing to give advice, discuss, and participate in the decision-making process. However, if the doctor-nurse relationship is not good, we may hesitate to ask the doctor for reasons or question the prescription. (N1)*

The sensitivity of doctors to their authority significantly impacts nurses' willingness to question their decisions. Doctors hold higher positions of power in clinical decision-making, especially when it comes to prescribing and AMS. This often leads nurses to hesitate in questioning doctors' decisions for fear of conflict or disrespecting their authority.

*Doctors are really touchy about their authority in AMS. Sometimes, they get annoyed with the supervision from clinical pharmacists and think they should be the ones making all the decisions, not to mention involving nurses. This could make the collaboration in healthcare even more strained. We (nurses) are hesitant to question their prescribing decisions, afraid it might cause conflict or make them think we don't respect their authority. So, in the end, we just keep quiet. (N4)*

The receptiveness of doctors towards nurses' suggestions played a crucial role in nurses' willingness to engage in AMS. If doctors respond negatively to nurses' suggestions, it can dampen their motivation to actively participate in AMS initiatives. In addition, the opinions of nursing peers within their working unit can influence nurses' attitudes towards AMS participation. Some interviewees mentioned being influenced by their nursing peers and adopting a conformist attitude towards engagement in AMS.

*The doctors showed little concern for our suggestions. Each time we offered advice, they failed to take it seriously, causing us to eventually give up. (N16)*

*When I get confused, I try to seek advice from more experienced colleagues. However, they often say that we just need to follow the doctor's orders. They have always done it this way. so, I just comply with it. (N17)*

The fear of being blamed is another significant factor that affects nurses' reluctance to actively engage in AMS practices. Nurses' concerns about their knowledge and abilities in AMS lead to feelings of insecurity and fear. They often fear providing incorrect advice or making mistakes that could potentially jeopardize patient safety. This fear of being held accountable for any negative outcomes further exacerbates their unwillingness to participate in AMS practices.

*After all, our knowledge about AMS is limited. What if we make a mistake? In such a situation, the doctor may hold us accountable, and the head nurse might criticize us as well. Who will ultimately be responsible? Sometimes, it may seem preferable not to take any action. (N2)*

#### 3.2.3. Theme 3: Limited Scopes and Absence of Standards in Nurses' Participation in AMS Practices

Incorporating the practice domain mentioned in the quantitative phase into the interview guideline, we investigated the perspectives and experiences of nurses regarding AMS practice. The interviewees highlighted that their involvement in AMS was constrained in terms of scope and form. In addition, the absence of guidelines and evaluation metrics further hindered their participation.

*(1) Sub-Theme 1: Limited Practice Scope and Form in AMS*. Nurses tend to engage in AMS practices that highly overlap with routine nursing practices. However, core nursing practices recommended by the ANA/CDC White Paper [34], such as multidisciplinary ward rounds, medication adjustments, and intravenous to oral transfers, are often excluded from their participation. Ten interviewees emphasized their exclusion from crucial AMS practices, including antimicrobial de-escalation and the switch from intravenous to oral therapy. These interviewees expressed uncertainty about the exact role and responsibilities in AMS. They hope to be more actively involved and provide advice on patient care. However, the power imbalance between doctors and nurses limits their participation in key practical decision-making for AMS. They feel like bystanders, unable to participate in important AMS practice decisions.

*As a nurse, I often find myself unsure about my exact role and responsibilities in AMS. We only follow the doctor's instructions and have no decision-making power. Although I will try my best to remind the doctors when skin tests are no longer effective and suggest rescheduling them accordingly, all decision-making power is in the hands of doctors. (N10)*

*We did not participate in key AMS practices such as antimicrobial de-escalation or the switch from intravenous to oral therapy. We feel like we're on the sidelines, watching the game but unable to play. (N3)*

*(2) Sub-Theme 2: Absence of Standardized Guidelines, Procedures, and Evaluation Indicators for Nurses' Participation*. The lack of standardized guidelines and procedures creates barriers to engagement. Nurses need a clear framework to follow in AMS, outlining the appropriate steps and actions to be taken in various situations. Without this clarity, nurses may not fully understand their responsibilities and may struggle to contribute effectively to AMS efforts.

*When I observed repeated infections or poor treatment outcomes in patients, this may be a sign of AMR. However, I often feel confused about how to accurately identify and report this situation, which may result in delays in taking appropriate measures to control and prevent AMR. If I had a guide or process to assist my work, I believe it would be better. (N14)*

This lack of standardized guidelines and procedures also extends to the evaluation of nurses' performance in AMS. Without established metrics to assess their effectiveness, nurses may not feel encouraged or supported. They may perceive no difference between being involved or not, leading to a lack of prioritization in AMS practice and insufficient attention being given to AMS.

*Is there a standard for participation or non-participation in AMS? How do we evaluate our AMS performance? Without standardised indicators, there is no difference between participating and not participating. So, we might as well not do it. We already have so much work, and this is not necessary. (N13)*

### 3.3. The Synthesis of Quantitative and Qualitative Results

**Figure 1:**
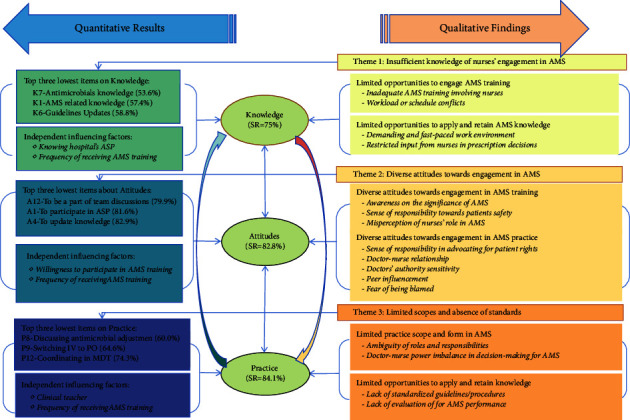
Joint display of the findings of the two-phase study. Note: IV to PO: intravenous to per oral; AMS: antimicrobial stewardship; ASP: antimicrobial stewardship programme; MDT: multidisciplinary team.

The qualitative phase of this study offers additional insights into the quantitative results and supplementary information on potential factors influencing nurses' engagement in AMS. In order to provide a comprehensive representation of the study findings, Figure 1 presents the combined results of the two-stage study in a graphical format.

## 4. Discussion

To our knowledge, this is the first mixed-methods study conducted in China that specifically assesses the KAP of nurses regarding AMS. In this study, we comprehensively assessed the KAP of Chinese nurses towards AMS and explored factors influencing these aspects. Our findings highlight the crucial need to enhance nurses' AMS knowledge, attitudes, and practice levels. Key influencing factors include awareness of the hospital's ASP, frequency of AMS training, willingness to participate in AMS training, and involvement in clinical teaching. The qualitative findings further reveal issues such as insufficient AMS knowledge, diverse attitudes towards AMS training and practice, and a limited scope and lack of guidelines in AMS practice.

Interestingly, only 57.5% of nurses in our study understood the importance and purpose of AMS, which is lower than the percentage reported by Abbas et al. (64.2%) [17]. This disparity in knowledge scores can be attributed to the difference in the availability of formal AMS training. In Abbas et al.'s study, nearly all nurses completed a mandatory online education program, while in our study, there was a lack of training and awareness, particularly in key areas such as antibiotic preparation and allergy testing. This highlights the importance of nurses continually updating their knowledge and adhering to the latest information and guidelines [6]. To bridge these knowledge gaps and promote responsible antimicrobial administration, hospitals must emphasize AMS training for nurses [6, 34]. This is further supported by the positive correlation between the frequency of receiving AMS education in the past year and participants' knowledge of AMS.

Providing comparative education is critical for healthcare professionals who are part of an AMS team or are interested in further involvement in AMS activities [35]. However, nurses do not always have the opportunity to participate in AMS training [36]. The qualitative research provides insight into this issue, revealing a lack of opportunities for nurses to engage in AMS training [36, 37]. Interviewees attributed this to insufficient AMS training provided by hospitals, with existing programmes primarily focusing on physicians, pharmacists, and microbiology technicians, neglecting the involvement of nurses. This reflects a significant oversight in recognizing the critical role nurses play in AMS [26]. This reflects a significant oversight in recognizing the critical role nurses play in AMS. To address this, there should be a greater emphasis on developing specialized curriculums for nurses [38], as recommended by the ANA/CDC White Paper [34]. In addition, implementing mandatory education programmes, similar to the one conducted in the Abbas et al. study, can ensure that all nurses receive comprehensive AMS training [17].

The lack of time to attend training was identified as a barrier for some nurses, as these sessions were typically scheduled during work hours, conflicting with nurses' heavy workloads and limiting their ability to participate. This indicates a lack of consideration for the practical constraints faced by nurses and further hinders their access to AMS training [35]. Innovative training models are essential to cater to the practical needs of nurses [39]. Continuing education can be delivered through various formats, including face-to-face sessions, online sessions, blended learning, workshops, and seminars [18, 35, 40, 41]. Among these options, online education stands out due to its numerous advantages like increased accessibility, flexibility, cost-effectiveness, and self-paced learning, which overcome barriers associated with traditional face-to-face education [40–43].

Several key issues affecting the application and retention of AMS knowledge were revealed based on the report of interviewees. The fast-paced work environment leaves them with limited time to consider the specific details of AMS, and their restricted input in prescription decisions further limits their ability to apply their knowledge in practice [44]. To address these challenges, a collaborative and inclusive approach to AMS is crucial [38]. This approach aligns with the principle of positive deviance, enabling nurses to leverage their required skills and knowledge [45]. Additionally, healthcare organizations should create a supportive environment where nurses are recognised as valuable contributors and provided with education and training to actively engage in AMS [46]. Encouraging open communication, interdisciplinary teamwork, and providing resources and time for ongoing learning are also helpful [38].

The study provides valuable insights into nurses' attitudes towards AMS and the factors influencing them. Our quantitative research findings suggest that nurses are generally positive about AMS. It is consistent with previous studies [16, 38, 47, 48], which highlight the shared recognition among healthcare professionals, including nurses, of the importance of judicious antimicrobial use in reducing the emergence and spread of AMR and improving patient outcomes [16]. However, qualitative analysis revealed diverse attitudes among nurses about engaging in both AMS training and practice. The underlying problem of nurses' lack of proactive participation in AMS training reflects challenges in education and awareness [49]. An inadequate comprehensive understanding of the importance of AMS and its implications for professional development and patient safety may be attributed to a lack of relevant training and education among nurses [50]. Furthermore, nurses may perceive AMS as being beyond their remit, diminishing their motivation to engage in AMS training. To address this issue, it is crucial to optimise the AMS training content targeted to fill knowledge gaps, as well as develop comprehensive AMS education and continuous professional development programmes [18, 51]. These initiatives should provide ample opportunities for professional growth and are designed to enhance nurses' understanding of AMS, clarify their role in AMS, and ultimately increase their willingness to participate in AMS training [13]. Additionally, this training should emphasize the connection between nursing contributions and AMS objectives, thereby enhancing their understanding of the importance of AMS participation [16].

The relationship between physicians and nurses is also crucial in shaping nurses' attitudes towards AMS [52, 53]. The study highlights the pressure nurses face due to doctors' authority sensitivity and emphasizes the importance of physicians recognizing nurses' competence and the need for supportive attitudes from healthcare colleagues. Improving physician-nurse relationships, fostering interprofessional collaboration and communication, and cultivating a positive work environment and culture are identified as significant factors in addressing these challenges [54]. Establishing interprofessional AMS committees or organising regular meetings to facilitate dialogue and cooperation among healthcare professionals is recommended as an effective strategy [55]. In addition, promoting a culture of continuous learning and knowledge sharing within healthcare institutions through peer-to-peer learning, mentorship programmes, and utilizing experts as resources can enhance nurses' understanding of AMS practices [56, 57].

Nurses' fear of being blamed can significantly impact their attitudes towards AMS practices. To address this, it is recommended to establish a supportive work environment that creates a sense of safety for nurses [58, 59]. Encouraging nurses to ask questions, seek clarification, and share their experiences, along with offering feedback and learning opportunities, can contribute to the ongoing improvement of their AMS knowledge and further motivate their active engagement in AMS practices [60]. By addressing these concerns and fostering a supportive environment, nurses can feel empowered to actively participate in AMS and contribute to its success [50].

Our study confirmed previous findings [38, 61] that nurses were confident in routine care practices, such as obtaining cultures before initiating antibiotics, assessing for antibiotic-associated adverse drug reactions, and educating patients and families about appropriate antibiotic use. However, we observed that nurses were less confident or hesitant when it came to participating in discussions regarding the adjustment of antimicrobial therapy, evaluating patients' swallowing function and oral medication ability, and coordinating activities with multidisciplinary professionals. This discrepancy highlights an imbalance in nurses' involvement and confidence in AMS [52, 61]. To address this issue, several factors must be considered, including inadequate training and guidance, workload pressures, and the need for improved communication and collaboration [54]. Addressing these issues will prepare nurses to participate more confidently in AMS, thereby improving the quality and safety of antibiotic use. The quantitative results demonstrating better AMS performance among clinical educators further validate this issue, which may be attributed to their advanced knowledge and skills.

To enhance nurses' participation in AMS practices, additional issues need to be addressed. One such issue is the lack of evaluation indicators for nurses' performance in AMS practices, which hampers their involvement [16]. Establishing clear assessment criteria will enable nurses to evaluate their effectiveness and drive their motivation and commitment [62]. Another issue is the lack of clear guidelines and standards, which can lead to nurses not fully understanding their responsibilities and not knowing how to effectively contribute to AMS efforts. Therefore, it is essential to develop comprehensive guidelines and standards that clearly define nurses' roles, responsibilities, and expectations in AMS practices [52]. These guidelines should provide clear guidance, empowering nurses to actively contribute to the antimicrobial decision-making process [62]. Proposed solutions to these problems should include encouraging nurses to actively participate in the rounding process and provide insights on various issues [50]. Tools like the SBAR (situation, background, assessment, recommendation) tool can facilitate effective communication between bedside nurses and healthcare providers [63, 64]. In addition, fostering a safe learning environment through group discussions, encouraging questions, and providing real-time education will reduce hierarchical perceptions and foster a shared understanding of the care management plan [54]. Furthermore, it is crucial that clinical leaders explicitly support and endorse the role of nurses in AMS [38]. This endorsement can further boost nurse confidence and participation in antibiotic management, leading to safer and more effective patient care [65].

This study has several limitations. Firstly, the self-reported questionnaire used in the study may have influenced respondents' answers due to subjectivity and potential social desirability bias. However, the explanatory sequential mixed methods employed in this study helped mitigate these limitations by validating the quantitative data through subsequent interviews. Despite the small qualitative sample size, the study achieved certain robust results. Second, the study was conducted in a specific province in China, which limits the generalizability of the findings to other regions within the country. Future research should consider expanding the survey area to include a more diverse sample. In addition, incorporating perspectives from other healthcare professionals, such as doctors, pharmacists, and hospital managers, would provide a more comprehensive understanding of nurses' involvement in AMS. Third, this study focused on nurses' attitudes in a medical setting where they are not authorized to prescribe medication. Therefore, generalizing the findings to other national or regional settings with different policies and medical contexts may be limited. Further research is needed to explore nurse KAP in various contexts to provide a more comprehensive understanding of the factors influencing AMS.

## 5. Implications for Nursing Management

Nursing managers should consider several key reforms to enhance nurses' participation in AMS initiatives. First, prioritising education and training programmes can improve nurses' knowledge and understanding of AMS principles and practices. Secondly, fostering collaboration and effective communication among healthcare professionals, including nurses, physicians, pharmacists, and other stakeholders, is essential to optimise antimicrobial use. Thirdly, nursing managers should ensure adequate staffing levels and workload management to allow nurses to dedicate sufficient time and attention to AMS activities without compromising patient care. Lastly, nursing managers should actively participate in policy formulation and implementation, advocating for the integration of AMS guidelines and standards into organisational policies and providing clear expectations for the participation of nurses in AMS.

## 6. Conclusions

The findings underscore the imperative to enhance nurses' understanding and engagement with AMS. Despite positive attitudes and practices, there's a noticeable knowledge gap that needs to be addressed. Influential factors included awareness of the hospital's ASP, frequency of AMS training, and willingness to participate in such training. Qualitative insights further elucidate the limited scope and lack of guidelines in AMS practice, reinforcing the need for more structured and frequent training initiatives. This study highlights the importance of targeted interventions to enhance AMS knowledge and engagement among nurses. It is essential to explicitly support the role of nurses in AMS, enhance education, acknowledge the practical constraints of nurses, and innovate training approaches. Fostering a supportive organisational culture, promoting multidisciplinary dialogue, ensuring an inclusive learning environment, and encouraging the development of AMS-integrated tools can empower nurses in AMS.

## Figures and Tables

**Table 1:** The three lowest and highest scoring items for the knowledge domain.

Items	Strongly disagree	Disagree	Not sure	Agree	Strongly agree
The top three lowest-scoring items					
K7. I know how to select the PH value and solvent volume in antibiotic configurations	4 (0.9)	58 (12.5)	153 (33.1)	186 (40.2)	62 (13.4)
K1. I know the purpose and significance of antimicrobial stewardship	8 (1.7)	67 (14.5)	122 (26.4)	209 (45.1)	57 (12.3)
K6. I know that *β* lactams have a high false-positive rate during a skin allergy test	3 (0.7)	42 (9.1)	146 (31.5)	200 (43.2)	72 (15.6)
The top three highest-scoring items					
K3. I know that culture specimens should be collected appropriately before using antimicrobials for treatment	8 (1.7)	33 (7.1)	52 (11.2)	209 (45.1)	161 (34.8)
K9. I understand time-based or concentration-based antibiotic administration	2 (0.4)	26 (5.6)	75 (16.2)	268 (57.9)	92 (19.9)
K5. I know the benefits of inquiring into antibiotic allergy history	1 (0.2)	36 (7.8)	81 (17.5)	245 (52.9)	100 (21.6)

**Table 2:** The three lowest and highest scoring items for the attitude domain.

Items	Strongly disagree	Disagree	Not sure	Agree	Strongly agree
The top three lowest-scoring items					
A12. Nurses should be part of team discussions regarding antimicrobial adjustments after 72 hours (withdrawal and de-escalation)	4 (0.9)	11 (2.4)	78 (16.8)	248 (53.6)	122 (26.3)
A1. Nurses should participate in antimicrobial stewardship programmes	4 (0.9)	9 (1.9)	72 (15.6)	244 (52.7)	134 (28.9)
A4. Nurses should be positive in acquiring up-to-date knowledge regarding antimicrobials and antimicrobial resistance	5 (1.1)	4 (0.9)	70 (15.1)	244 (52.7)	140 (30.2)
The top three highest-scoring items					
A8. Nurses should be aware of the importance of infection prevention and control measures (hand hygiene, standard precautions, etc.) to prevent nosocomial infections	3 (0.6)	6 (1.3)	39 (8.4)	252 (54.4)	163 (35.2)
A3. Nurses should be aware that rational and prudent use of antimicrobials can delay and reduce the development of multidrug-resistant bacteria	3 (0.6)	7 (1.5)	41 (8.9)	243 (52.5)	169 (36.5)
A11. Nurses should promptly identify incorrect antimicrobial orders and inform the physician when such orders are received	3 (0.6)	5 (1.1)	44 (9.5)	245 (52.9)	166 (35.9)

**Table 3:** The three lowest and highest scoring items for the practice domain.

Items	Never	Rarely	Sometimes	Often	Always
The top three lowest-scoring items					
P8. Participating in discussions regarding antimicrobial adjustment (de-escalation or discontinuation)	25 (5.4)	20 (4.3)	140 (30.3)	163 (35.2)	115 (24.8)
P9. Assessing patients' swallowing function and ability to take oral medications and recommending switching to oral administration as necessary	26 (5.6)	16 (3.5)	122 (26.3)	173 (37.4)	126 (27.2)
P12. Coordinating and discussing antimicrobial therapy with multiple professionals	11 (2.4)	17 (3.7)	91 (19.6)	181 (39.1)	163 (35.2)
The top three highest-scoring items					
P1. Informing patients of proper retention methods and precautions when collecting sputum and urine samples	0 (0.0)	9 (1.9)	22 (4.8)	145 (31.3)	287 (62.0)
P7. Monitoring and reporting adverse reactions to antimicrobial treatment	0 (0.0)	7 (1.5)	32 (6.9)	142 (30.7)	282 (60.9)
P5. Providing antimicrobials without delay, adhering to the appropriate drip rate, and documenting the administration	0 (0.0)	9 (1.9)	29 (6.3)	187 (40.4)	238 (51.4)

**Table 4:** Univariate analysis of AMS knowledge, attitudes, and practices of nurses with different demographic characteristics (*N* = 463).

Variables	*n* (%)	Knowledge		Attitudes		Practices	
		Mean (SD)	*t*/*F* (*P*)	Mean (SD)	*t*/*F* (*P*)	Mean (SD)	*t*/*F* (*P*)
Sex			−1.33 (0.18)		−0.28 (0.78)		−1.45 (0.15)
Male	31 (93.3)	61.77 (10.72)		57.52 (10.37)		56.58 (9.03)	
Female	432 (6.7)	64.50 (11.03)		57.97 (8.70)		58.94 (8.77)	
Age (years)			**2.84 (0.02)** ^*∗*^		1.83 (0.12)		0.34 (0.85)
20–30	141 (30.4)	63.43 (10.83)		58.65 (8.35)		58.48 (8.30)	
31–35	196 (42.3)	63.75 (11.70)		56.93 (9.38)		58.54 (9.23)	
36–40	92 (19.9)	64.77 (9.42)		58.05 (8.09)		59.71 (8.55)	
41–45	16 (3.5)	69.06 (11.13)		58.81 (9.45)		59.13 (8.55)	
>45	18 (3.9)	71.00 (10.10)		61.88 (7.81)		58.89 (9.85)	
Education level			0.28 (0.76)		1.07 (0.35)		0.51 (0.60)
Junior college or lower	16 (3.5)	65.31 (11.92)		56.38 (7.88)		60.94 (9.77)	
Bachelor degree	425 (91.8)	64.21 (11.13)		57.87 (8.72)		58.69 (8.83)	
Graduate degree	22 (4.7)	65.77 (8.35)		60.32 (8.28)		59 (7.65)	
Professional title			**3.71 (0.01)** ^*∗*^		1.52 (0.21)		1.01 (0.39)
Junior nurse	64 (13.8)	61.75 (11.00)		58.63 (7.84)		57.14 (8.59)	
Senior nurse	221 (47.7)	63.99 (11.32)		57.63 (8.80)		58.87 (9.01)	
Supervisor nurse	163 (35.2)	65.11 (10.41)		57.67 (9.28)		59.17 (8.67)	
Cochief nurse and higher	15 (3.2)	71.53 (10.16)		62.33(6.49)		60.33 (7.68)	
Clinical teacher			**−3.54 (<0.01)** ^*∗*^		−0.29 (0.77)		−**3.17 (<0.01)**^*∗*^
No	217 (46.9)	62.41 (10.98)		57.81 (8.43)		57.42 (9.01)	
Yes	246 (53.1)	66.00 (10.81)		58.05 (9.14)		59.99 (8.44)	
Knowing that the hospital has an ASP			**−6.83 (<0.01)** ^*∗*^		**−2.84 (0.01)** ^*∗*^		**−4.84 (<0.01)** ^*∗*^
No	146 (31.5)	59.40 (11.26)		56.25 (8.61)		55.94 (9.35)	
Yes	317 (68.5)	66.58 (10.16)		58.71 (8.80)		60.09 (8.22)	
Nursing experience (years)			**3.02 (0.01)** ^*∗*^		1.14 (0.34)		1.47 (0.20)
<1	24 (5.2)	58.33 (11.19)		57.54 (8.13)		54.21 (9.85)	
1–5	103 (22.2)	63.77 (9.86)		58.39 (7.94)		58.63 (7.89)	
6–10	154 (33.3)	63.90 (11.96)		57.72 (9.91)		59.31 (8.83)	
11–15	117 (25.3)	64.85 (11.01)		56.92 (8.57)		58.91 (9.33)	
16–20	34 (7.3)	65.74 (9.02)		58.62 (7.81)		59.47 (8.07)	
>20	31 (6.7)	69.32 (9.83)		60.90 (7.95)		59.00 (9.00)	
Willingness to undergo AMS training			**−2.05 (0.04)** ^*∗*^		**−3.51 (<0.01)** ^*∗*^		−1.66 (0.11)
No	33 (7.1)	60.55 (12.30)		52.82 (11.96)		56.33 (8.97)	
Yes	430 (92.9)	64.61 (10.88)		58.33 (8.41)		58.97 (8.77)	
Frequency of receiving AMS education in the past year			**25.41 (<0.01)** ^*∗*^		**12.15 (<0.01)** ^*∗*^		**20.61 (<0.01)** ^*∗*^
0	178 (38.4)	59.77 (10.28)		56.13 (8.97)		55.83 (9.36)	
1	134 (28.9)	64.65 (10.67)		56.66 (9.65)		58.17 (8.59)	
2	71 (15.3)	67.63 (10.18)		59.92 (6.63)		61.59 (6.36)	
≥3	80 (17.3)	70.95 (9.45)		62.35 (6.64)		63.89 (6.55)	
Department			**2.43 (0.05)**		**2.77 (0.03)** ^*∗*^		**4.95 (<0.01)** ^*∗*^
Intensive care unit	76 (16.4)	61.16 (11.98)		55.84 (8.62)		56.11 (9.24)	
Internal medicine department	162 (35.0)	64.23 (9.97)		57.14 (8.50)		57.63 (8.59)	
Department of surgery	169 (36.5)	65.05 (11.85)		59.08 (9.40)		60.77 (8.96)	
Department of emergency	26 (5.6)	66.23 (9.79)		58.96 (6.70)		59.73 (7.29)	
Department of gynaecology	30 (6.5)	67.03 (8.73)		60.27 (7.96)		59.80 (6.35)	

Notes: ^*∗*^*P* value's significance at the 0.05 level. Abbreviations. SD, standard deviation. AMS, antimicrobial stewardship. ASP, antimicrobial stewardship programme.

**Table 5:** Multivariate analysis of nurses' knowledge, attitudes, and practices regarding AMS.

Domains	Independent variables	*β*	SE	*β*′	t	*P*
Knowledge	Constant	46.341	6.669	—	6.949	<0.001
	Knowing the ASP in their hospital	3.872	1.160	0.163	3.337	<0.001
	Frequency of receiving AMS education in the past year	2.955	0.471	0.296	6.270	<0.001

Attitudes	Constant	46.202	5.634	—	8.201	<0.001
	Willingness to undergo AMS training	3.658	1.582	0.107	2.312	0.021
	Frequency of receiving AMS education in the past year	1.701	0.398	0.213	4.273	<0.001

Practices	Constant	50.001	5.430	—	9.208	<0.001
	Clinical teacher	2.084	0.914	0.118	2.280	0.023
	Frequency of receiving AMS education in the past year	2.212	0.384	0.278	5.765	<0.001

Notes: knowledge: *F* = 8.166, *P* < 0.001, *R*^*2*^ = 0.191, adjusted *R*^*2*^ = 0.168. Attitudes: *F* = 3.662, *P* < 0.001, *R*^*2*^ = 0.096, adjusted *R*^*2*^ = 0.070. Practices: *F* = 6.498, *P* < 0.001, *R*^*2*^ = 0.158, adjusted *R*^*2*^ = 0.134. Abbreviations. AMS, antimicrobial stewardship. ASP, antimicrobial stewardship programme.

## Data Availability

All data from the study are available from the corresponding author upon reasonable request.

## Supplementary Materials

Supplementary Materials Please refer to the following supplementary files for the relevant study guidance and checklists: Supplementary File 1: the STROBE statement guiding the quantitative study, while Supplementary File 2: the COREQ checklist guiding the qualitative study. Supplementary File 3: the GRAMMS Checklist. Supplementary File 4: the interview guidelines for the quantitative phase. Furthermore, detailed information on all items of nurses' knowledge, attitudes, and practices (KAP) on antimicrobial stewardship (AMS) can be found in Supplementary Table 1. The characteristics of the 17 interviewees in the qualitative phase are presented in Supplementary Table 2.
