# Supplementary material for: Knowledge, Attitudes, and Practices of Bedside Nurses regarding Antimicrobial Stewardship in China: An Explanatory Sequential Mixed Methods Study
Source: J Nurs Manag. 2023 Nov 22;2023:9059920. doi: 10.1155/2023/9059920 (PMC11919010; doi:10.1155/2023/9059920)
Supplement: Supplementary Materials — Please refer to the following supplementary files for the relevant study guidance and checklists: Supplementary File 1: the STROBE statement guiding the quantitative study, while Supplementary File 2: the COREQ checklist guiding the qualitative study. Supplementary File 3: the GRAMMS Checklist. Supplementary File 4: the interview guidelines for the quantitative phase. Furthermore, detailed information on all items of nurses' knowledge, attitudes, and practices (KAP) on antimicrobial stewardship (AMS) can be found in Supplementary Table 1. The characteristics of the 17 interviewees in the qualitative phase are presented in Supplementary Table 2. [file 9059920.f1.zip › Supplementary File 3 (2).docx]

*Manuscript Title: Knowledge, attitudes, and practices of bedside nurses regarding antimicrobial stewardship in China: An explanatory sequential mixed methods study*

**Supplementary File 3** Good Reporting of a Mixed Methods Study (GRAMMS) checklist

| **Good** **reporting** **of** **a** **mixed-methods** **study** **(GRAMMS)** **guidance** | **Guidance** **met?** **(Section:** **page**  **number** **in** **the** **manuscript)** | |
| --- | --- | --- |
| Describe the justification for using a mixed methods approach to the research question | Yes  Introduction: 3,4 | |
| Describe the design in terms of the purpose, priority and sequence of methods | Yes  Introduction: 3,4  Methods: 4 | |
| Describe each method in terms of sampling, data collection and analysis | Yes  Participants and Sampling: 4,5  Data collection: 5-7  Data analysis: 7-8 | |
| Describe where integration has occurred, how it has occurred and who has participated in it | Yes  Data integration: 9  Results: 20 (Figure1) | |
| Describe any limitation of one method associated with the present of the other method | Yes  Discussion: 24, 25 |  |
| Describe any insights gained from mixing or integrating methods | Yes  Discussion: 21-26 | |

*Ref:* *O'Cathain* *A,* *Murphy* *E,* *Nicholl* *J.* *The* *quality* *of* *mixed* *methods* *studies* *in* *health* *services* *research.* *J* *Health* *Serv* *Res* *Policy.* *2008;13:* *92-98*
