# Supplementary material for: Knowledge, Attitudes, and Practices of Bedside Nurses regarding Antimicrobial Stewardship in China: An Explanatory Sequential Mixed Methods Study
Source: J Nurs Manag. 2023 Nov 22;2023:9059920. doi: 10.1155/2023/9059920 (PMC11919010; doi:10.1155/2023/9059920)
Supplement: Supplementary Materials — Please refer to the following supplementary files for the relevant study guidance and checklists: Supplementary File 1: the STROBE statement guiding the quantitative study, while Supplementary File 2: the COREQ checklist guiding the qualitative study. Supplementary File 3: the GRAMMS Checklist. Supplementary File 4: the interview guidelines for the quantitative phase. Furthermore, detailed information on all items of nurses' knowledge, attitudes, and practices (KAP) on antimicrobial stewardship (AMS) can be found in Supplementary Table 1. The characteristics of the 17 interviewees in the qualitative phase are presented in Supplementary Table 2. [file 9059920.f1.zip › Supplementary Table 1 KAP score of nurses.docx]

Supplementary Table 1 Knowledge, attitudes, and practice scores of nurses regarding antimicrobial stewardship (AMS); n, (%).

| **Domains** | **Items** | **Strongly Disagree** | **Disagree** | **Not Sure** | **Agree** | **Strongly Agree** |
| --- | --- | --- | --- | --- | --- | --- |
| **Knowledge** | | | | | | |
|  | K1. I know the purpose and significance of AMS. | 8  (1.7) | 67  (14.5) | 122 (26.4) | 209 (45.1) | 57  (12.3) |
|  | K2. I know that AMS requires multidisciplinary teams. | 8  (1.7) | 43  (9.3) | 77  (16.6) | 232 (50.1) | 103 (22.3) |
|  | K3. I know that culture specimens should be collected appropriately before using antimicrobials for treatment. | 8  (1.7) | 33  (7.1) | 52  (11.2) | 209 (45.1) | 161 (34.8) |
|  | K4. I know the diagnostic criteria for multidrug-resistant bacterial infection. | 5  (1.1) | 39  (8.4) | 132 (28.5) | 210 (45.4) | 77  (16.6) |
|  | K5. I know the benefits of inquiring into antibiotic allergy history. | 1  (0.2) | 36  (7.8) | 81  (17.5) | 245 (52.9) | 100 (21.6) |
|  | K6. I know that β lactams have a high false-positive rate during a skin allergy test. | 3  (0.7) | 42  (9.1) | 146 (31.5) | 200 (43.2) | 72  (15.6) |
|  | K7. I know how to select the pH value and solvent volume in antibiotic configurations. | 4  (0.9) | 58  (12.5) | 153 (33.1) | 186 (40.2) | 62  (13.4) |
|  | K8. I know the drugs that are incompatible with commonly used antibiotics. | 1  (0.2) | 28  (6.1) | 84  (18.1) | 251 (54.2) | 99  (21.4) |
|  | K9. I understand time-based or concentration-based antibiotic administration. | 2  (0.4) | 26  (5.6) | 75  (16.2) | 268 (57.9) | 92  (19.9) |
|  | K10. I know the adverse reactions and specific clinical manifestations of antibiotics. | 2  (0.4) | 29  (6.3) | 131 (28.3) | 239 (51.6) | 62  (13.4) |
|  | K11. I know that broad-spectrum antibiotics are more susceptible to bacterial resistance. | 7  (1.5) | 27  (5.8) | 108 (23.3) | 248 (53.6) | 73  (15.8) |
|  | K12. I know the potential risks associated with the use of antibiotics over a long period. | 11  (2.4) | 38  (8.2) | 116 (25.1) | 225 (48.6) | 73  (15.8) |
|  | K13. I know that patients may benefit from switching from intravenous to oral therapy as soon as they are clinically stable. | 6  (1.3) | 23  (5.0) | 108 (23.3) | 257 (55.5) | 69  (14.9) |
|  | K14. I know the requirements for switching from intravenous to oral therapy. | 5  (1.1) | 27  (5.8) | 111 (24.0) | 250 (54.0) | 70  (15.1) |
|  | K15. I know the indications for antimicrobial treatment in patients with asymptomatic bacteriuria. | 8  (1.7) | 39  (8.4) | 131 (28.3) | 223 (48.2) | 62  (13.4) |
| **Attitudes** | | | | | | |
|  | **Items** | **Strongly Disagree** | **Disagree** | **Not Sure** | **Agree** | **Strongly Agree** |
|  | A1. Nurses should participate in antimicrobial stewardship programs. | 4  (0.9) | 9  (1.9) | 72  (15.6) | 244 (52.7) | 134 (28.9) |
|  | A2. Nurses should be responsible for implementing antimicrobial practices and acting as a bridge between patients and other healthcare professionals. | 4  (0.9) | 7  (1.5) | 64  (13.8) | 255 (55.1) | 133 (28.7) |
|  | A3. Nurses should be aware that rational and prudent use of antimicrobials can delay and reduce the development of multidrug-resistant bacteria. | 3  (0.6) | 7  (1.5) | 41  (8.9) | 243 (52.5) | 169 (36.5) |
|  | A4. Nurses should be positive in acquiring up-to-date knowledge regarding antimicrobials and antimicrobial resistance. | 5  (1.1） | 4  (0.9) | 70 (15.1） | 244 (52.7) | 140 (30.2) |
|  | A5. Nurses should be responsible for collecting culture specimens early and correctly before administering antimicrobials. | 4  (0.9) | 6  (1.3) | 51  (11.0) | 257 (55.5) | 145 (31.3) |
|  | A6. Nurses should screen for incorrectly reported antimicrobial allergies in patients by asking about antimicrobial allergy history. | 3  (0.6) | 6  (1.3) | 59  (12.7) | 254 (54.9) | 141 (30.5) |
|  | A7. Nurses should recognize special infection symptoms and signs when receiving hospitalized patients and take isolation precautions if necessary. | 3  (0.6) | 7  (1.5) | 50  (10.8) | 258 (55.7) | 145 (31.3) |
|  | A8. Nurses should be aware of the importance of infection prevention and control measures (hand hygiene, standard precautions, etc.) to prevent nosocomial infections. | 3  (0.6) | 6  (1.3) | 39  (8.4) | 252 (54.4) | 163 (35.2) |
|  | A9. Nurses should review susceptibility test results for pathogenic bacteria, identify patients with multidrug-resistant organisms and follow standard precautions. | 4  (0.9) | 7  (1.5) | 55  (11.9) | 260 (56.2) | 137 (29.6) |
|  | A10. Nurses should carefully monitor adverse reactions to antibiotics and inform doctors and pharmacists as soon as possible if they occur. | 2  (0.4) | 7  (1.5) | 44  (9.5) | 251 (54.2) | 159 (34.3) |
|  | A11. Nurses should promptly identify incorrect antimicrobial orders and inform the physician when such orders are received. | 3  (0.6) | 5  (1.1) | 44  (9.5) | 245  (52.9) | 166 (35.9) |
|  | A12. Nurses should be part of team discussions regarding antimicrobial adjustments after 72 hours (withdrawal and de-escalation). | 4  (0.9) | 11  (2.4) | 78  (16.8) | 248 (53.6) | 122 (26.3) |
|  | A13. Nurses should facilitate an early and reasonable transition from intravenous to oral therapy by observing the patient’s condition and communicating with the physician. | 5  (1.1) | 8  (1.7) | 65  (14.0) | 255 (55.1) | 130 (28.1) |
|  | A14. Nurses should provide patients with education on the rational use of antimicrobials. | 3  (0.6) | 7  (1.5) | 54  (11.7) | 256 (55.3) | 143 (30.9) |
| **Practices** | | | | | | |
|  | **Items** | **Never** | **Rarely** | **Sometimes** | **Often** | **Always** |
|  | P1. Informing patients of proper retention methods and precautions when collecting sputum and urine samples | 0  (0.0) | 9  (1.9) | 22  (4.8) | 145 (31.3) | 287 (62.0) |
|  | P2. Consulting and discussing with prescribers regarding the solvent, dosage, route of administration, duration, etc., when in doubt | 4  (0.9) | 16  (3.5) | 66  (14.3) | 178 (38.4) | 199 (43.0) |
|  | P3. Inquiring and recording antimicrobial allergy history, including the drug name, strength, route, date of administration, allergic symptoms, the time when symptoms emerged, and subsequent treatment with related antibiotics | 1  (0.2) | 16  (3.5) | 45  (9.7) | 184 (39.7) | 217 (46.9) |
|  | P4. Determining culture and drug susceptibility results and informing the physician of the results | 2  (0.4) | 20  (4.3) | 66  (14.3) | 177 (38.2) | 198 (42.8) |
|  | P5. Providing antimicrobials without delay, adhering to the appropriate drip rate, and documenting the administration | 0  (0.0) | 9  (1.9) | 29  (6.3) | 187 (40.4) | 238 (51.4) |
|  | P6. Informing patients and family members of symptoms of antimicrobial allergies or adverse reactions that may occur because of their administration | 2  (0.4) | 8  (1.7) | 49  (10.6) | 173 (37.4) | 231 (49.9) |
|  | P7. Monitoring and reporting adverse reactions to antimicrobial treatment | 0  (0.0) | 7  (1.5) | 32 (6.9) | 142 (30.7) | 282 (60.9) |
|  | P8. Participating in discussions regarding antimicrobial adjustment (de-escalation or discontinuation) | 25  (5.4) | 20  (4.3) | 140 (30.3) | 163 (35.2) | 115 (24.8) |
|  | P9. Assessing patients’ swallowing function and ability to take oral medications and recommending switching to oral administration as necessary | 26  (5.6) | 16  (3.5) | 122 (26.3) | 173 (37.4) | 126 (27.2) |
|  | P10. Providing education to patients and their families about oral antimicrobial agents and antimicrobial resistance | 8  (1.7) | 16  (3.5) | 68  (14.7) | 197 (42.5) | 174 (37.6) |
|  | P11. Coordinating and discussing antimicrobial therapy and antimicrobial resistance with colleagues | 4  (0.9) | 20  (4.3) | 53  (11.4) | 185 (40.0) | 201 (43.4) |
|  | P12. Coordinating and discussing antimicrobial therapy with multiple professions | 11  (2.4) | 17  (3.7) | 91  (19.6) | 181 (39.1) | 163 (35.2) |
