# Supplementary material for: Knowledge, Attitudes, and Practices of Bedside Nurses regarding Antimicrobial Stewardship in China: An Explanatory Sequential Mixed Methods Study
Source: J Nurs Manag. 2023 Nov 22;2023:9059920. doi: 10.1155/2023/9059920 (PMC11919010; doi:10.1155/2023/9059920)
Supplement: Supplementary Materials — Please refer to the following supplementary files for the relevant study guidance and checklists: Supplementary File 1: the STROBE statement guiding the quantitative study, while Supplementary File 2: the COREQ checklist guiding the qualitative study. Supplementary File 3: the GRAMMS Checklist. Supplementary File 4: the interview guidelines for the quantitative phase. Furthermore, detailed information on all items of nurses' knowledge, attitudes, and practices (KAP) on antimicrobial stewardship (AMS) can be found in Supplementary Table 1. The characteristics of the 17 interviewees in the qualitative phase are presented in Supplementary Table 2. [file 9059920.f1.zip › Supplementary Table 2 .docx]

*Manuscript Title: Knowledge, attitudes, and practices of bedside nurses regarding antimicrobial stewardship in China: An explanatory sequential mixed methods study*

**Supplementary Table 2** Seventeen interviewees’ Demographics Characteristic

| Characteristic | Participants (n=17) |
| --- | --- |
|  |  |
| Gender |  |
| Male | 1(5.88) |
| Female | 16(94.12) |
| Age(year) |  |
| 18-35 | 7(41.18) |
| 36-45 | 7(41.18) |
| ≥46 | 3(17.65) |
| Professional title |  |
| Junior title | 6(35.29) |
| Intermediate title | 8(47.06) |
| Senior title | 3(17.65) |
| Education level |  |
| Junior college or lower | 4(23.53) |
| Bachelor degree | 9(52.94) |
| Graduate degree | 4(23.53) |
| Nursing experience, Year |  |
| ＜10 | 5(29.41) |
| 11-20 | 9(52.94) |
| ＞20 | 3(17.65) |
| Clinical Department |  |
| Intensive Care Unit(ICU) | 3(17.65) |
| Emergency Department | 1(5.88) |
| Department of Respiratory Medicine | 1(5.88) |
| Department of Cardiothoracic Surgery | 1(5.88) |
| Department of Neurology | 1(5.88) |
| Department of General Surgery | 1(5.88) |
| Department of Pediatric Internal Medicine | 1(5.88) |
| Department of Neurosurgery | 2(11.76) |
| Department of Infectious Diseases | 2(11.76) |
| Department of Paediatrics Surgery | 2(11.76) |
| Department of Hematology and Oncology | 2(11.76) |
| Both direct nursing and management |  |
| Yes | 5(29.41) |
| No | 12(70.59) |
