# Supplementary material for: Knowledge, Attitudes, and Practices of Bedside Nurses regarding Antimicrobial Stewardship in China: An Explanatory Sequential Mixed Methods Study
Source: J Nurs Manag. 2023 Nov 22;2023:9059920. doi: 10.1155/2023/9059920 (PMC11919010; doi:10.1155/2023/9059920)
Supplement: Supplementary Materials — Please refer to the following supplementary files for the relevant study guidance and checklists: Supplementary File 1: the STROBE statement guiding the quantitative study, while Supplementary File 2: the COREQ checklist guiding the qualitative study. Supplementary File 3: the GRAMMS Checklist. Supplementary File 4: the interview guidelines for the quantitative phase. Furthermore, detailed information on all items of nurses' knowledge, attitudes, and practices (KAP) on antimicrobial stewardship (AMS) can be found in Supplementary Table 1. The characteristics of the 17 interviewees in the qualitative phase are presented in Supplementary Table 2. [file 9059920.f1.zip › Supplementary File 2 (2).doc]

*Manuscript Title: Knowledge, attitudes, and practices of bedside nurses regarding antimicrobial stewardship in China: An explanatory sequential mixed methods study*

**Supplementary File 2** Consolidated Criteria for Reporting Qualitative Research (COREQ)

| **No. Item** | **Guide questions/description** | **Reported on Page #** |
| --- | --- | --- |
| **Domain 1: Research team and reﬂexivity** |  |  |
| *Personal Characteristics* |  |  |
| 1. Interviewer/facilitator | Which author/s conducted the inter view or focus group? | Author #1 Page 6-7 |
| 2. Credentials | What were the researcher’s credentials? E.g. PhD, MD | Author #1 & 5 & 6 & 8 & 9: PhD Candidates  Author #2 & 3 & 4 & 7 & 10  : MD  Title page |
| 3. Occupation | What was their occupation at the time of the study? | Title page |
| 4. Gender | Was the researcher male or female? | Female |
| 5. Experience and training | What experience or training did the researcher have? | Page 7 |
| *Relationship with participants* |  |  |
| 6. Relationship established | Was a relationship established prior to study commencement? | Yes Page 6-7 |
| 7. Participant knowledge of the interviewer | What did the participants know about the researcher? e.g. personal goals, reasons for doing the research | Page 5-7 |
| 8. Interviewer characteristics | What characteristics were reported about the inter viewer/facilitator? e.g. Bias, assumptions, reasons and interests in the research topic | Page 5-7 |

| **Domain 2: study design** |  |  |
| --- | --- | --- |
| *Theoretical framework* |  |  |
| 9. Methodological orientation and Theory | What methodological orientation was stated to underpin the study? e.g. grounded theory, discourse analysis, ethnography, phenomenology, content analysis | Page 7，8  phenomenology |
| *Participant selection* |  |  |
| 10. Sampling | How were participants selected? e.g. purposive, convenience, consecutive, snowball | Page 5 |
| 11. Method of approach | How were participants approached? e.g. face-to-face, telephone, mail, email | Page 6  Face-to-face |
| 12. Sample size | How many participants were in the study? | 17; Page 13 |
| 13. Non-participation | How many people refused to participate or dropped out? Reasons? | No, All participants were voluntary and there were no sample dropouts.  Page 5,13 |
| *Setting* |  |  |
| 14. Setting of data collection | Where was the data collected? e.g. home, clinic, workplace | Page 6 |
| 15. Presence of non-participants | Was anyone else present besides the participants and researchers? | None, Page 6,7 |
| 16. Description of sample | What are the important characteristics of the sample? e.g. demographic data, date | Page 13; Supplementary Table 1 |
| *Data collection* |  |  |
| 17. Interview guide | Were questions, prompts, guides provided by the authors? Was it pilot tested? | Yes, Page 6  Supplementary File 4 |
| 18. Repeat interviews | Were repeat inter views carried out? If yes, how many? | Page 7, 3times |
| 19. Audio/visual recording | Did the research use audio or visual recording to collect the data? | Page 7 |
| 20. Field notes | Were ﬁeld notes made during and/or after the inter view or focus group? | Yes, P7 |
| 21. Duration | What was the duration of the inter views or focus group? | Page 7 30-50min |
| 22. Data saturation | Was data saturation discussed? | Yes P7 |
| 23. Transcripts returned | Were transcripts returned to participants for comment and/or correction? | Yes P8 |
| **Domain 3: analysis and ﬁndings** |  |  |
| *Data analysis* |  |  |
| 24. Number of data coders | How many data coders coded the data? | Page 8 |
| 25. Description of the coding tree | Did authors provide a description of the coding tree? | Page 8 |
| 26. Derivation of themes | Were themes identiﬁed in advance or derived from the data? | Derived from the data Page 8 |
| 27. Software | What software, if applicable, was used to manage the data? | Page 7, 8  NVivo V.12 (QSR International, Doncaster, Australia) |
| 28. Participant checking | Did participants provide feedback on the ﬁndings? | P8 |
| *Reporting* |  |  |
| 29. Quotations presented | Were participant quotations presented to illustrate the themes/ﬁndings? Was each quotation identiﬁed? e.g. participant number | Page 13-19 |
| 30. Data and ﬁndings consistent | Was there consistency between the data presented and the ﬁndings? | Yes P13-20 |
| 31. Clarity of major themes | Were major themes clearly presented in the ﬁndings? | Yes P13-20 |
| 32. Clarity of minor themes | Is there a description of diverse cases or discussion of minor themes? | Yes P22-25 |

*Ref: Tong A, Sainsbury P, Craig J. Consolidated criteria for reporting qualitative research (COREQ): a 32-item checklist for interviews and focus groups. International Journal for Quality in Health Care. 2007. 19(6): 349 – 357*
