# Supplementary material for: Knowledge, Attitudes, and Practices of Bedside Nurses regarding Antimicrobial Stewardship in China: An Explanatory Sequential Mixed Methods Study
Source: J Nurs Manag. 2023 Nov 22;2023:9059920. doi: 10.1155/2023/9059920 (PMC11919010; doi:10.1155/2023/9059920)
Supplement: Supplementary Materials — Please refer to the following supplementary files for the relevant study guidance and checklists: Supplementary File 1: the STROBE statement guiding the quantitative study, while Supplementary File 2: the COREQ checklist guiding the qualitative study. Supplementary File 3: the GRAMMS Checklist. Supplementary File 4: the interview guidelines for the quantitative phase. Furthermore, detailed information on all items of nurses' knowledge, attitudes, and practices (KAP) on antimicrobial stewardship (AMS) can be found in Supplementary Table 1. The characteristics of the 17 interviewees in the qualitative phase are presented in Supplementary Table 2. [file 9059920.f1.zip › supplementary File 4 (2).doc]

*Manuscript Title: Knowledge, attitudes, and practices of bedside nurses regarding antimicrobial stewardship in China: An explanatory sequential mixed methods study*

**Supplementary File 4** Qualitative interviews guideline

Thank you for agreeing to participate in this interview regarding nurses' involvement in antimicrobial drug management knowledge, attitudes, and practices. We hope to gain insight into the role and challenges of nurses in antimicrobial stewardship, as well as their attitudes and level of engagement.

**Knowledge：**

*1. What is your current understanding of antimicrobial stewardship?*

*2. Can you explain what antimicrobial stewardship entails?*

*3. Who comprises the antimicrobial stewardship team in your hospital? How do you perceive the role of nurses within this team? Could you provide further details on this?*

*4. What are your views on nurses' involvement in antimicrobial stewardship?*

*5.What are some of the challenges you have encountered in your experience with antimicrobial stewardship?*

*6.What challenges do you think exist in other nurses’ engagement with AMS?*

*7.What do you believe are the potential benefits of nurses participating in antimicrobial stewardship initiatives?*

*8.How do you stay up-to-date with the latest information and guidelines related to antimicrobial stewardship?*

*9.Could you discuss any specific examples of your involvement in antimicrobial stewardship activities?*

*10. Based on our survey, the majority of nurses express a lack of knowledge in AMS. What is your perspective on this matter?*

*6. Does your hospital or department provide training on antimicrobial drug management? Would you be willing to participate? Is participation in the training mandatory? Can you elaborate on the specific content of the training program?*

**Atititudes**

*1. If the hospital requests your participation in antibacterial drug management related training, would you be willing to participate?*

*2. Please elaborate on your perspective regarding nurses' involvement in antimicrobial drug management.*

*3.What do you think are the factors that influence nurses' participation in antimicrobial stewardship initiatives?*

*4. Can you provide examples to illustrate the attitudes of your colleagues towards nurses participating in antimicrobial drug management?*

*5. Considering our previous investigation, which indicates that most nurses are willing to participate in AMS, what is your opinion on this finding?*

*6. In your opinion, what are some reasons that may cause hesitation among nurses in participating in antimicrobial stewardship activities?*

*7. Do the leaders of your department support nurses' engagement? Do you have any related policy documents? Are there any reward or punishment measures for this?*

*8. What is your department head or Matron's attitude towards this matter?*

*9.How do you see the role of nurses in antimicrobial stewardship evolving in the future?*

**Practice**

*1. In your opinion, what are the scopes in which nurses can actively participate in antimicrobial stewardship initiatives?*

*2. Regarding the recommended practices from foreign countries, could you provide an in-depth discussion on which ones you believe align with your views and which ones you disagree with? The practices include:*

*(1) Identifying symptoms and signs of infection*

*(2) Accurately assessing patients' history of antibiotic allergies*

*(3) Collecting appropriate culture specimens prior to antimicrobial administration*

*(4) Participating in discussions about de-escalation based on patient assessment*

*(5) Participating in discussions about transitioning from intravenous to oral antibiotics*

*(6) Reviewing preliminary and final culture results*

*(7) Providing antibiotic education to patients and their families*

*3. Have doctors invited you to participate in discussions on antibiotic usage?*

*4. Do you take part in doctor's rounds? Please explain the specific actions or roles you undertake during these rounds.*

*5. Are there any nurses in your workplace who play an active role in antimicrobial stewardship? Could you provide examples of their involvement and the specific tasks they perform?*

*6. Are there situations in your work where you question a doctor's decision regarding antibiotic usage? What prompted you to do this or not?*

*7. In your opinion, what factors either facilitate or hinder your engagement in antimicrobial stewardship activities?*

*8.What do you believe are the potential benefits of increased collaboration between nurses and other healthcare professionals in antimicrobial stewardship?*

*Thank you for sharing your valuable insights and experiences with us. We greatly appreciate your perspective on nurses' involvement in antimicrobial management, which will help us better understand this important topic. If we have any further questions or need clarification, we may reach out to you for a follow-up interview. Thank you once again.*
